# Application of Mössbauer Spectroscopy to the Study of Iron Phase States in Products of Magnetization Roasting of Ferruginous Manganese Ore and Concentrate

**DOI:** 10.3390/ma19143017

**Published:** 2026-07-13

**Authors:** Alibek Baisanov, Sailaubai Baisanov, Nina Vorobkalo, Askhat Akuov, Yerulan Samuratov, Inkar Mussina

**Affiliations:** 1 Laboratory of Pyrometallurgical Processes, Zh. Abishev Chemical-Metallurgical Institute, Karaganda 100009, Kazakhstan; alibekbaisanov-chmi@mail.ru (A.B.); akuov.am@mail.ru (A.A.); yerulansamuratov2@gmail.com (Y.S.); pirometall.hmi09@mail.ru (I.M.); 2 Laboratory of Metallurgical Melts, Zh. Abishev Chemical-Metallurgical Institute, Karaganda 100009, Kazakhstan

**Keywords:** ferruginous manganese ore, ferruginous manganese concentrate, magnetization roasting, magnetic separation, Mössbauer spectroscopy, iron phase state, substituted magnetite, Mn/Fe ratio, low-grade manganese raw materials

## Abstract

**Highlights:**

**What are the main findings?**

- Magnetization roasting increased the Mn/Fe ratio of non-magnetic products.
- Magnetite-type spinels were preferentially concentrated in magnetic fractions.
- Residual iron occurred as hematite-like and paramagnetic Fe components.

**What are the implications of the main findings?**

- Mössbauer spectroscopy clarifies iron phase transformations.
- Iron removal depends on both spinel formation and spinel distribution.
- Iron phase analysis explains the mechanism of roasting–magnetic beneficiation.

**Abstract:**

Magnetization roasting followed by magnetic separation can upgrade ferruginous manganese raw materials, but its efficiency depends on the iron phase state after roasting. This study clarified the phase state and distribution of iron in magnetic and non-magnetic products obtained from ferruginous manganese ore and concentrate after magnetization roasting and dry magnetic separation. The materials were roasted at 600–650 °C using Shubarkol coal as a solid reducing agent and then separated at a magnetic field strength of 1.2 kOe. Chemical analysis, X-ray diffraction, and Mössbauer spectroscopy were used to relate separation results to iron phase states. The Mn/Fe ratio in the non-magnetic products increased from 3.92 to 10.87 for the ore and from 2.98 to 23.99 for the concentrate, while 76.25% and 88.26% of iron were transferred to the corresponding magnetic fractions. Mössbauer spectroscopy showed that magnetite-type spinel components, including Fe_3_O_4_ and substituted (Fe_1−x_M_x_)_3_O_4_, were mainly associated with the magnetic products. Hematite-like and paramagnetic Fe^3+^/Fe^2+^ components were also detected, indicating incomplete conversion of iron-bearing phases into magnetite-type spinels. The Mössbauer-area-weighted fraction of iron in magnetite-type spinel phases was 76.5% for the ore and 64.36% for the concentrate. These results explain the different separation behavior after roasting–magnetic treatment.

## 1. Introduction

Manganese is a strategically important element for the production of steel and manganese ferroalloys, primarily ferromanganese and silicomanganese [1]. Its importance is reflected in its inclusion in the U.S. Critical Minerals List, while in the European Union manganese is classified as a critical raw material, and high-purity battery-grade manganese is additionally recognized as a strategic raw material [2,3,4]. In iron and steel metallurgy, manganese acts as a desulfurizing, deoxidizing, and alloying element, improving the strength, hardness, wear resistance, toughness, and weldability of steels [3]. According to the U.S. Geological Survey, global manganese mine production in 2024 was approximately 20.0 million tonnes on a Mn-content basis, while global reserves were estimated at approximately 1.7 billion tonnes; South Africa accounts for the largest share of global manganese resources [2,5].

The quality of manganese raw materials is determined not only by Mn content but also by ore mineralogy, iron, silica and phosphorus contents, and the Mn/Fe ratio. The Mn/Fe ratio is crucial for the production of commercial manganese-containing products. Based on the manganese-to-iron ratio (Mn/Fe), manganese ores are classified into the following types [1,6,7,8]:

- Manganese ores (Mn/Fe > 6–7), with a Mn content of more than 35%;
- Ferruginous manganese ores (Mn/Fe ~ 1), with a Mn content of 15–35%;
- Manganiferous iron ores (Mn/Fe < 1), with a Mn content of 5–10%.

Globally, high-grade manganese ores are predominantly concentrated in South Africa, Gabon, Australia, and Brazil. Medium- and low-grade manganese ores, including ferruginous manganese varieties, are widespread in India, China, Kazakhstan, Ukraine, and several other countries. In particular, India is characterized by a significant proportion of ferruginous manganese ores, while in China, more than 73% of manganese ores are ferruginous manganese ores with a low Mn/Fe mass ratio of <3 [4,9].

Large reserves of low-grade manganese ores occur in many countries worldwide. These deposits are only partially used in the production of manganese ferroalloys. These ores contain considerable amounts of iron, which results in a low Mn/Fe ratio. Iron occurs partly as discrete hematite and goethite grains, whereas the remaining iron is incorporated into manganese-bearing minerals [10]. For such raw materials, preliminary removal of the iron-bearing component and an increase in the Mn/Fe ratio to a level suitable for ferroalloy processing are essential [11].

Kazakhstan is not among the world’s largest manganese producers, but it has an industrially significant manganese raw material base. According to national industrial statistics, in 2023, the country produced 930.7 thousand tonnes of raw manganese ore, corresponding to 176.0 thousand tonnes of Mn, while manganese concentrate production reached 431.3 thousand tonnes. The main manganese deposits are concentrated in the Ulytau and Karaganda regions (Atasu, Zhezdy, and Ushkatyn deposit groups), which makes the processing of low-grade and ferruginous manganese ores in Kazakhstan particularly relevant [12,13,14].

The involvement of ferruginous manganese ores in metallurgical processing is especially relevant as rich oxidized ores are gradually depleted and attention shifts to more complex manganese-bearing raw materials [15,16,17,18,19,20]. However, previous studies on low-grade and ferruginous manganese ores have shown that beneficiation efficiency is largely controlled by mineral composition, the form of iron occurrence, and the degree of intergrowth between manganese- and iron-bearing phases [8,10,21,22]. Since fine dissemination and complex Mn–Fe intergrowth limit the selectivity of conventional physical separation, magnetization roasting followed by magnetic separation is considered a feasible approach for increasing the Mn/Fe ratio [23,24,25]. During roasting, weakly magnetic or non-magnetic iron-bearing phases can be transformed into magnetite-type phases and removed into the magnetic fraction, whereas the manganese-enriched product is mainly concentrated in the non-magnetic fraction. However, interactions between manganese and iron oxides may also lead to mixed spinel-type phases and solid solutions, including Mn_x_Fe_3−x_O_4_-type oxides, which can affect magnetic response and reduce Mn–Fe separation selectivity [23]. Similar complex Mn–Fe oxide and spinel associations, including jacobsite-bearing assemblages, have been reported for the Ushkatyn-III deposit [16,17]. Therefore, the technological result of roasting–magnetic processing cannot be fully explained only by bulk chemical composition or the final Mn/Fe ratio; it is necessary to determine the phase state of iron in both magnetic and non-magnetic products.

At present, the practical application of roasting–magnetic beneficiation to ferruginous manganese raw materials is limited by several technological bottlenecks. The reduction conditions must be sufficient to transform weakly magnetic or non-magnetic iron-bearing phases into magnetite-type phases, but excessive or non-uniform reduction may promote Fe^2+^-bearing non-spinel phases, mixed Mn–Fe spinels, or incompletely converted hematite-like components [23,26]. In addition, the magnetic response of the roasted product depends not only on the amount of magnetite-type phases formed, but also on their liberation, grain size, fine intergrowth with manganese-bearing minerals, and cation substitution in the spinel lattice [22,23]. Recent studies on thermal processing and sintering of iron-bearing ores also show that heat distribution, particle-size effects, and phase formation strongly affect product quality and process stability [27,28]. For ferruginous manganese ores, these issues are especially important because Mn–Fe oxide and spinel phases may have overlapping diffraction reflections and different magnetic behavior.

Although X-ray diffraction and chemical analysis provide information on crystalline phases and elemental composition, they do not always reliably distinguish iron-bearing phases with overlapping reflections, defect-rich structures, mixed Fe–Mn spinels, or different valence and magnetic states of iron. Mössbauer spectroscopy is particularly suitable for this purpose because its hyperfine parameters, including isomer shift, quadrupole splitting, effective hyperfine magnetic field, and relative spectral area, allow magnetically ordered and paramagnetic iron states to be identified and clarify the formation of magnetite, non-stoichiometric or substituted magnetite-type phases, hematite, and other iron-bearing components. Previous studies of burned ferrous manganese ores using nuclear gamma-resonance spectroscopy showed that this method can clarify the phase structure and reduction behavior of iron-bearing manganese ores [29,30].

The aim of this study was to clarify the phase state of iron in the products obtained after magnetization roasting and dry magnetic separation of ferruginous manganese ore and ferruginous manganese concentrate. The study focused on determining the distribution of iron-bearing phases between magnetic and non-magnetic fractions, identifying magnetically ordered and paramagnetic iron states using Mössbauer spectroscopy, assessing the formation of magnetite-type, substituted spinel-type, hematite-type, and other iron-bearing components, and relating these phase transformations to the increase in the Mn/Fe ratio after roasting–magnetic treatment.

The novelty of the work lies in linking the redistribution of Mössbauer-resolved iron-bearing phases between magnetic and non-magnetic products with the increase in the Mn/Fe ratio after magnetization roasting and dry magnetic separation. This provides a phase-level explanation of separation efficiency that cannot be obtained from bulk chemical composition alone.

## 2. Materials and Methods

### 2.1. Raw Materials and Reducing Agent

Ferruginous manganese ore and ferruginous manganese concentrate (Zhanaarka district, Ulytau region, Kazakhstan) were used as the initial materials in this study. The materials were characterized by low Mn/Fe ratios and a complex mineral composition, which makes direct physical separation of manganese- and iron-bearing components difficult. Therefore, magnetization roasting followed by dry magnetic separation was applied to increase the Mn/Fe ratio and to redistribute iron-bearing phases into the magnetic fraction. The chemical compositions of the initial materials are presented in Table 1.

Low-ash long-flame coal from the Shubarkol deposit, Karaganda region, Kazakhstan, was used as a solid reducing agent during magnetization roasting. This coal was selected because of its relatively high volatile matter content and its ability to generate a reducing atmosphere during heating. The reducing conditions in the charge were formed by solid carbon and by gaseous products released during coal pyrolysis and combustion, mainly CO, H_2_, and CH_4_. These gases can participate in the reduction of iron oxides to magnetite-type phases, while solid carbon can contribute to contact reduction of iron-bearing minerals.

The main technical characteristics, elemental composition, ash composition, and gas composition of the reducing agent are presented in Table 2.

### 2.2. Magnetization Roasting and Dry Magnetic Separation

**Figure 1:**
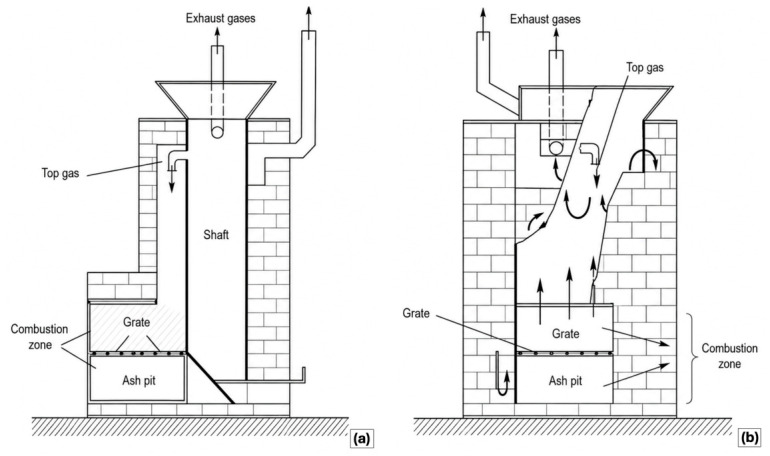
Schematic diagram of the externally heated chamber-type shaft furnace used for magnetization roasting of ferruginous manganese raw materials: (**a**) furnace structure; (**b**) gas-flow pattern in the combustion and roasting zones.

Magnetization roasting of ferruginous manganese ore and ferruginous manganese concentrate was carried out in a custom-built externally heated chamber-type shaft furnace. The furnace included a shaft for the ore–reductant charge, a combustion zone equipped with a grate and an ash pit, and gas channels that provided the movement of hot gases along the roasting chamber. External heating of the working chamber was achieved through combustion of volatile products released from long-flame coal. A schematic representation of the furnace structure and gas-flow pattern is shown in Figure 1.

The ore–reductant mixture was prepared from ferruginous manganese material with a particle size of 0–10 mm and lump Shubarkol coal with a particle size of 10–80 mm. The raw material-to-coal mass ratio was 1:0.3. Roasting was carried out under reducing conditions for 8–12 h at a target temperature of 600–650 °C. After roasting, the coarse carbonaceous residue formed from Shubarkol coal was removed by screening, and the roasted material was subjected to dry magnetic separation.

Dry magnetic separation was carried out using a 120-T-type dry magnetic separator (Yueyang, China) for strongly magnetic materials. A nominal magnetic field strength of 1.2 kOe was used. Operationally, the separator was operated at a transformer current of 10–12 A. As a result, magnetic and non-magnetic fractions were obtained separately from the roasted ferruginous manganese ore and from the roasted ferruginous manganese concentrate.

Four separation products were selected for further study: the magnetic fraction of roasted ferruginous manganese ore, the non-magnetic fraction of roasted ferruginous manganese ore, the magnetic fraction of roasted ferruginous manganese concentrate, and the non-magnetic fraction of roasted ferruginous manganese concentrate. This sample set made it possible to compare the phase state of iron depending on both the initial raw material type and the magnetic behavior of the products after roasting–magnetic treatment.

### 2.3. Chemical and X-Ray Diffraction Analyses

The chemical composition of the initial materials, reducing agent, and magnetic separation products was determined in the certified analytical laboratory of the Zh. Abishev Chemical-Metallurgical Institute, Karaganda, Kazakhstan. The contents of the major components were determined using a MAX-GVM X-ray fluorescence spectrometer (SPA “Spectron”, St. Petersburg, Russia) and by photometric chemical analysis. The carbon content was determined separately by high-temperature combustion of samples in an oxygen atmosphere using a dedicated analytical unit operated by the institute staff.

X-ray diffraction analysis was performed to determine the crystalline phase composition of the studied separation products. The analysis was carried out using a Rigaku MiniFlex 600 X-ray diffractometer (Rigaku Corporation, Akishima, Tokyo, Japan) with Cu-Kα radiation. Phase identification was performed using the PDF-5 database. The X-ray diffraction data were used together with Mössbauer spectroscopy results to compare the crystalline phase composition with the iron phase states identified from hyperfine parameters.

### 2.4. Mössbauer Spectroscopy

Mössbauer spectroscopy was used to determine the phase state, magnetic ordering, and local crystallochemical environment of iron in the products of magnetization roasting and dry magnetic separation. The spectra were recorded at 293 K using an SM-2201 Mössbauer spectrometer (NUST MISIS, Moscow, Russia). A ^57^Co source in a chromium matrix with an activity of 100 mCi was used as the γ-radiation source.

The spectra were processed using least-squares fitting. The isomer shift values, δ, are given relative to α-Fe. During spectral processing, the following hyperfine parameters were determined: isomer shift δ, quadrupole splitting ΔEQ, effective hyperfine magnetic field H_eff_, and relative spectral area S. The uncertainties were ±0.03 mm/s for the isomer shift and quadrupole splitting, ±5 kOe for the effective hyperfine magnetic field, and ±3–5% for the relative spectral areas.

The Mössbauer spectra were interpreted taking into account the magnetically ordered and paramagnetic states of iron. Magnetically ordered components were used to identify phases such as Fe_3_O_4_, α-Fe_2_O_3_, and substituted magnetite of the composition (Fe_1−x_M_x_)_3_O_4_, where M may represent Mn, Mg, or other elements that isomorphically substitute for iron in the crystal lattice.

The paramagnetic spectral components were interpreted by comparison with reported hyperfine parameters of Fe^3+^- and Fe^2+^-bearing phases. Fe^3+^ doublets were assigned to residual non-spinel ferric components, including highly dispersed ferric oxide components or ferric sites in silicate phases. Fe^2+^ doublets were attributed to ferrous non-spinel components, including Fe_1−x_O-like and silicate-like local environments.

### 2.5. Calculation Procedures

The Mn/Fe ratio was calculated as the ratio of the total manganese content to the total iron content in the corresponding material or separation product.

The distribution of manganese and iron between the magnetic and non-magnetic fractions was calculated to evaluate the separation efficiency after magnetization roasting. The distribution of component *i* in product *j* was calculated using the product yield and the content of the corresponding component (1):

(1) $$D_{i,j}=\frac{\gamma_{j}\cdot C_{i,j}}{C_{i,0}}$$

where *D_i,_*_j_ is the distribution of component i in product *j*, %; *γ_j_* is the yield of the corresponding product *j*, %; *C_i,j_* is the content of component *i* in product *j,* wt.%; and *C_i,_*_0_ is the content of the same component in the initial material, wt.%.

The obtained Mössbauer parameters were used to evaluate the distribution of iron among magnetite-type spinel and non-spinel components in the roasted separation products. In this work, the Mössbauer-area-weighted fraction of iron present in magnetite-type spinel phases was defined as the fraction of total iron represented by magnetite Fe_3_O_4_ and substituted magnetite-type spinel components (Fe_1−x_M_x_)_3_O_4_. This value was calculated from the relative Mössbauer spectral areas, taking into account the yield and total iron content of each separation product, according to Equation (2):

(2) $$D_{sp}=\;\frac{\gamma_{m\;}\cdot \;C_{Fe,m}\;\cdot \;S_{sp,\;\;m}\;+\gamma_{nm\;}\cdot \;C_{Fe,nm}\;\cdot \;S_{sp,\;nm}}{\gamma_{m\;}\cdot \;C_{Fe,m}\;+\gamma_{nm\;}\cdot \;C_{Fe,nm}\;}$$

where *D_sp_* is the Mössbauer-area-weighted fraction of iron present in magnetite-type spinel phases, %; *γ_m_* and *γ_nm_* are the yields of the magnetic and non-magnetic fractions, %; *C_Fe,m_* and *C_Fe,nm_* are the total iron contents in the magnetic and non-magnetic fractions, wt.%; and *S_sp,m_* and *S_sp,nm_* are the total relative Mössbauer spectral areas of magnetite-type spinel components in the corresponding fractions, %.

The value *S_sp_* included the spectral areas assigned to Fe_3_O_4_ and (Fe_1−x_M_x_)_3_O_4_. Hematite-like α-Fe_2_O_3_ components, residual Fe^3+^ non-spinel components, and paramagnetic Fe^2+^ components were not included in this calculation. Therefore, *D_sp_* should be interpreted not as saturation magnetization, magnetic susceptibility, phase mass fraction, or volume fraction, but as a Mössbauer-area-weighted estimate of the fraction of iron present in magnetite-type spinel components in the roasted separation products.

## 3. Results and Discussion

### 3.1. Results of Magnetization Roasting and Dry Magnetic Separation

The products obtained after magnetization roasting and dry magnetic separation were evaluated by yield, chemical composition, Mn/Fe ratio, and the distribution of manganese and iron between the magnetic and non-magnetic fractions. The characteristics of the products of dry magnetic separation of roasted ferruginous manganese ore are given in Table 3.

At a magnetic field strength of 1.2 kOe, the yield of the magnetic fraction was 41.40%, whereas the yield of the non-magnetic fraction was 58.60%. The Mn/Fe ratio in the magnetic fraction was 1.75, while in the non-magnetic fraction it increased to 10.87. Compared with the initial ore, where the Mn/Fe ratio was 3.92, the non-magnetic product was substantially enriched in manganese relative to iron. This indicates that roasting promoted the formation of magnetically recoverable iron-bearing phases and their subsequent removal into the magnetic fraction.

For roasted ferruginous manganese concentrate, the separation effect was more pronounced (Table 4). The magnetic fraction accounted for 18.58% of the product yield. Its Mn/Fe ratio was only 0.16, confirming the high iron concentration in the magnetic product. The non-magnetic fraction accounted for 81.42% of the total yield. As a result, the Mn/Fe ratio increased from 2.98 in the original concentrate to 23.99 in the non-magnetic fraction.

The calculated distribution of manganese and iron between the magnetic and non-magnetic fractions is presented in Table 5.

In the case of ferruginous manganese ore, 76.25% of the iron was transferred to the magnetic fraction, while 65.86% of the manganese remained in the non-magnetic fraction. For ferruginous manganese concentrate, 88.26% of the iron was concentrated in the magnetic fraction, while 95.29% of the manganese remained in the non-magnetic fraction. Thus, roasting followed by dry magnetic separation resulted in a selective redistribution of iron and manganese: iron was predominantly transferred to the magnetic fraction, while manganese remained primarily in the non-magnetic product.

The relatively high SiO_2_ content in the non-magnetic ore fraction does not exclude its metallurgical use. For silicomanganese and other Si–Mn-bearing ferroalloy production, silicon-bearing components are required in the charge together with manganese-bearing materials. Therefore, the obtained Mn-enriched non-magnetic products may be considered as potential feed materials for silicomanganese or other Si–Mn-bearing alloy production, provided that the final charge composition and impurity limits are adjusted for the selected smelting process. This interpretation is consistent with the importance of Fe–Si and Al–Si–Mn phase relations in the formation of silicon-containing ferroalloy systems [18,19].

The higher selectivity observed for the concentrate can be related to differences in the initial chemical and mineral composition of the materials. The concentrate initially contained more iron and had a lower Mn/Fe ratio, which made the effect of iron removal more pronounced. At the same time, the high manganese recovery in the non-magnetic fraction indicates that manganese-bearing phases were not substantially transferred into the magnetic product under the selected roasting and separation conditions.

The lower iron contents in the non-magnetic fractions, 1.82 wt.% Fe for the roasted ore product and 1.63 wt.% Fe for the roasted concentrate product, resulted in a weaker Mössbauer effect and a lower signal-to-noise ratio compared with the magnetic fractions. Nevertheless, the fitted spectra allowed the main magnetically ordered and paramagnetic iron components to be distinguished.

### 3.2. Phase Composition and Iron Phase States of Roasted Ferruginous Manganese Ore

**Figure 2:**
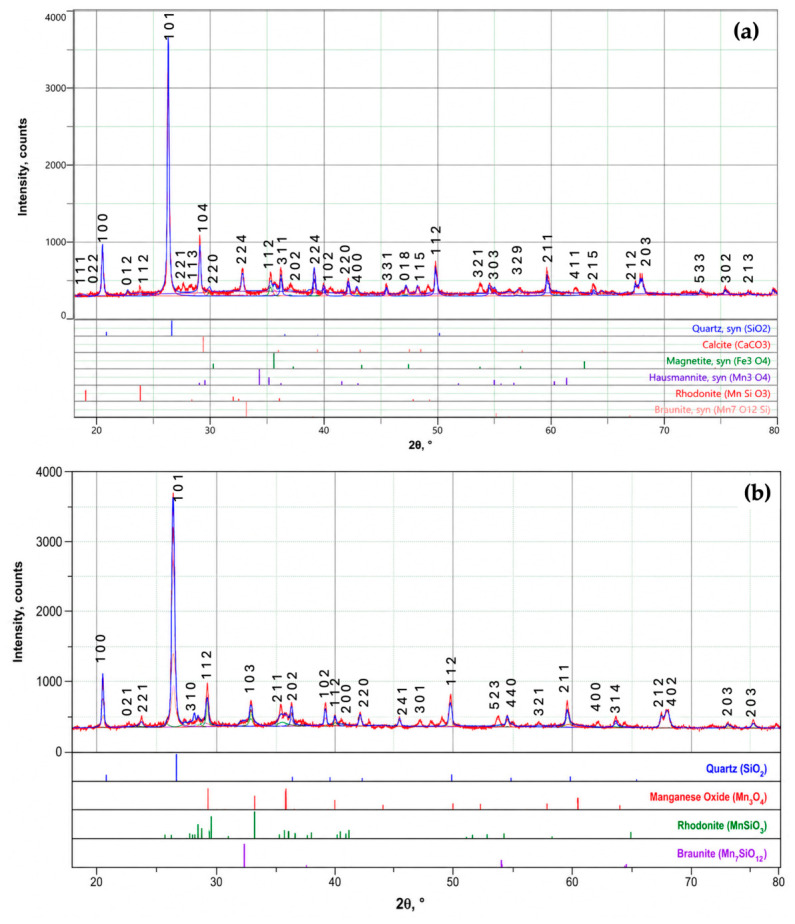
X-ray diffraction patterns of the products obtained from roasted ferruginous manganese ore: (**a**) magnetic fraction; (**b**) non-magnetic fraction.

**Figure 3:**
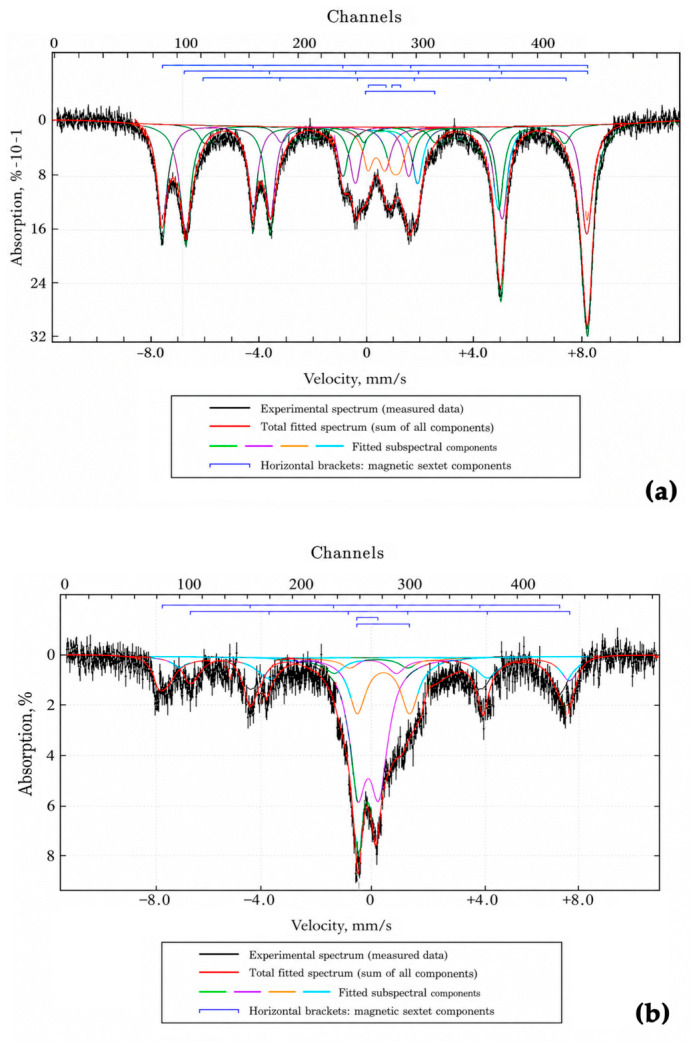
Mössbauer spectra of the products obtained from roasted ferruginous manganese ore: (**a**) magnetic fraction; (**b**) non-magnetic fraction.

The phase composition and phase states of iron in the magnetic and non-magnetic fractions obtained from roasted ferruginous manganese ore are shown in Figure 2 and Figure 3. The Mössbauer hyperfine parameters obtained from spectral fitting are summarized in Table 6.

X-ray diffraction analysis showed that both fractions of the roasted ore are complex multiphase products. The magnetic fraction contained manganese-bearing oxide and silicate phases, quartz, carbonate phases, and magnetite-type spinel components. The presence of magnetite-type phases is consistent with the transfer of most iron into the magnetic fraction. At the same time, the magnetic fraction still contained a noticeable amount of manganese, which indicates either mechanical entrainment of manganese-bearing minerals or their association with iron-bearing spinel phases.

The non-magnetic fraction was dominated by manganese-bearing oxide and silicate phases together with quartz and other gangue minerals. Magnetite-type reflections were weak or overlapped with reflections of manganese oxides and Mn–Fe spinel-type phases. Therefore, X-ray diffraction alone is insufficient for reliable identification of the iron-bearing components in this material, especially because magnetite, hausmannite, jacobsite, and substituted Mn–Fe spinels may have partially overlapping diffraction lines.

Thus, X-ray diffraction established the general oxide–silicate–spinel phase assemblage of the roasted ore products, whereas Mössbauer spectroscopy resolved the iron-bearing part of this assemblage according to magnetic ordering and local Fe environment. This combined interpretation is important because several Mn–Fe oxide and spinel phases have overlapping diffraction reflections, while their Fe hyperfine parameters differ in the Mössbauer spectra.

Mössbauer spectroscopy clarified the iron phase states in these products. In the magnetic fraction, the spectrum was dominated by three magnetically ordered components with effective hyperfine magnetic fields of 487, 460, and 413 kOe and relative spectral areas of 37, 42, and 7%, respectively. These components were assigned to substituted magnetite-type spinel phases of the general composition (Fe_1−x_M_x_)_3_O_4_. The presence of three magnetic components instead of the two sublattices typical of stoichiometric magnetite indicates a disturbed local environment of iron atoms. This disturbance is most likely caused by partial substitution of iron by manganese, magnesium, or other cations present in the raw material.

Minor paramagnetic components were also detected in the magnetic fraction. The component with δ = 1.05 mm/s and ΔEQ = 0.30 mm/s was interpreted as a Fe^2+^ paramagnetic component with Fe_1−x_O-like hyperfine parameters. Other minor doublets may correspond to iron in sulfide-, sulfate-, or silicate-like local environments. Since these components accounted for only a small part of the spectral area, they did not determine the magnetic behavior of the product.

In the non-magnetic fraction of roasted ore, Mössbauer spectroscopy also revealed magnetically ordered magnetite-type spinel components. The sextets with effective hyperfine magnetic fields of 487 and 463 kOe accounted for 27 and 19% of the relative spectral area, respectively. This indicates that part of the spinel-type iron remained in the non-magnetic product. Such behavior may be caused by incomplete liberation of iron-bearing grains, fine intergrowth with manganese-bearing silicate or oxide phases, or reduced magnetic response of substituted spinels compared with stoichiometric magnetite.

The paramagnetic part of the spectrum of the non-magnetic ore fraction was more pronounced than that of the magnetic fraction. The doublet with δ = 0.39 mm/s and ΔEQ = 0.85 mm/s was assigned to a residual Fe^3+^ non-spinel component. Taking into account the multiphase oxide–silicate composition of the non-magnetic ore fraction, this component may be associated with dispersed ferric oxide sites or Fe^3+^ incorporated in silicate phases. The doublet with δ = 0.99 mm/s and ΔEQ = 2.11 mm/s may correspond to Fe^2+^ in a silicate-like local environment. These data show that part of the iron remained in weakly magnetic or non-magnetic forms after roasting.

For ferruginous manganese ore, magnetite-type spinel components accounted for 86% of the relative Mössbauer spectral area in the magnetic fraction and 46% in the non-magnetic fraction. Therefore, the Mössbauer-area-weighted fraction of iron present in magnetite-type spinel phases was calculated using the yield and total iron content of both separation products:

$$D_{sp}=\frac{41.40\cdot 8.27\cdot 86+58.60\cdot 1.82\cdot 46}{41.40\cdot 8.27+58.60\cdot 1.82}=76.5\%$$

Thus, approximately 76.5% of iron in the roasted ore products occurred in magnetite-type spinel phases. This confirms that magnetization roasting produced a substantial amount of magnetically recoverable iron-bearing spinel components. However, the detection of spinel-type components in the non-magnetic fraction indicates that separation was incomplete, most likely because part of the iron-bearing phases remained finely intergrown with manganese-bearing minerals.

### 3.3. Phase Composition and Iron Phase States of Roasted Ferruginous Manganese Concentrate

**Figure 4:**
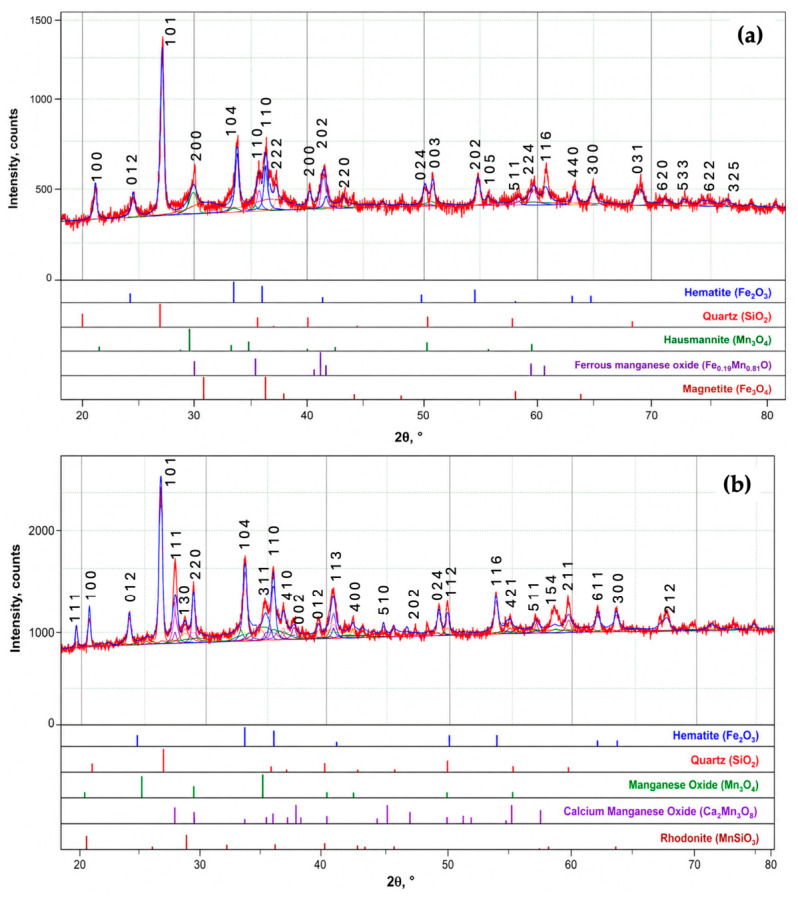
X-ray diffraction patterns of the products obtained from roasted ferruginous manganese concentrate: (**a**) magnetic fraction; (**b**) non-magnetic fraction.

**Figure 5:**
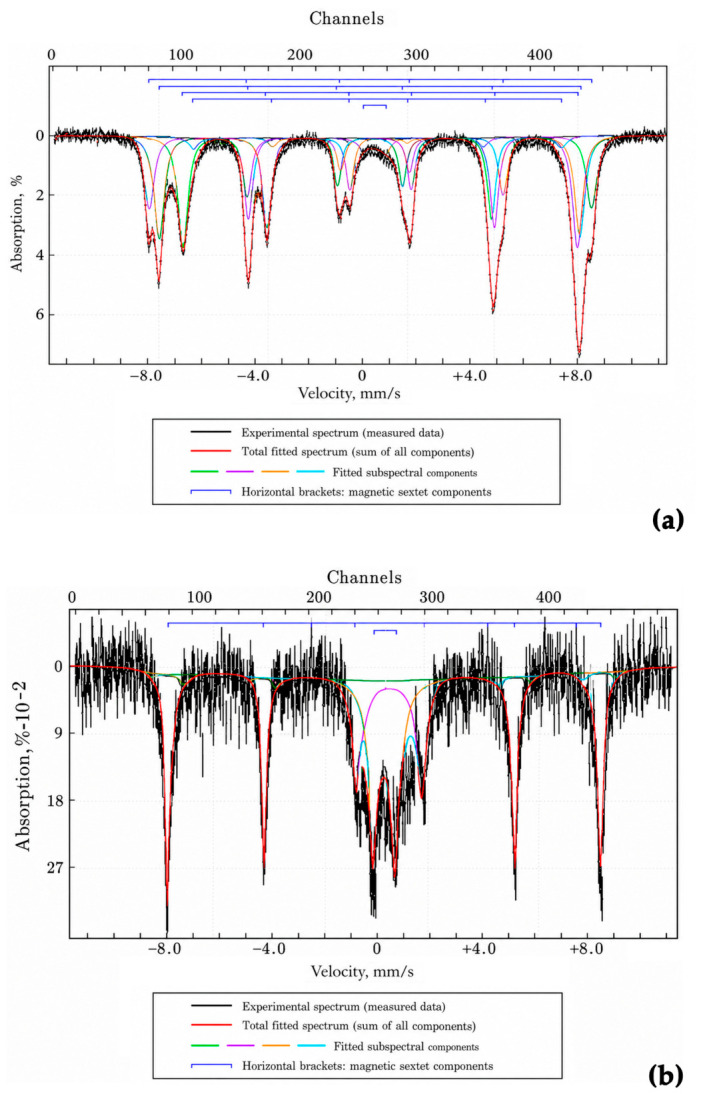
Mössbauer spectra of the products obtained from roasted ferruginous manganese concentrate: (**a**) magnetic fraction; (**b**) non-magnetic fraction.

The X-ray diffraction patterns and Mössbauer spectra of the magnetic and non-magnetic fractions obtained from roasted ferruginous manganese concentrate are shown in Figure 4 and Figure 5. The Mössbauer hyperfine parameters obtained from spectral fitting are summarized in Table 7.

X-ray diffraction analysis of the magnetic fraction of roasted concentrate showed the presence of iron-bearing oxide phases, including hematite and magnetite-type spinel components, together with manganese oxides, quartz, and associated crystalline phases. The detection of magnetite-type phases is consistent with the high iron content of the magnetic product and with the preferential transfer of iron into this fraction. The presence of hematite indicates that reduction of all ferric iron-bearing phases to magnetite-type phases was incomplete under the selected roasting conditions.

The XRD detection of hematite and magnetite-type spinel reflections is consistent with the Mössbauer spectrum of the magnetic fraction, which contained both a hematite-like sextet and substituted magnetite-type spinel components. The sextet with H_eff_ = 511 kOe, δ = 0.37 mm/s, and ΔEQ = −0.18 mm/s accounted for 24% of the relative spectral area and was interpreted as a hematite-like α-Fe_2_O_3_ component. The slightly lower effective hyperfine field compared with ideal hematite may be associated with lattice distortion, fine dispersion, or partial substitution of iron by other cations.

The main part of the spectrum was represented by substituted magnetite-type spinel components. Three sextets with H_eff_ = 487, 456, and 425 kOe and relative spectral areas of 33, 36, and 4%, respectively, were assigned to (Fe_1−x_Mx)_3_O_4_. Their total relative spectral area was 73%. This indicates that magnetite-type spinel components made the main contribution to the magnetic behavior of the concentrate product. As in the roasted ore, the presence of several magnetic components reflects differences in the local environment of iron atoms and may be associated with partial substitution by Mn, Mg, or other cations.

The non-magnetic fraction of roasted concentrate had the highest Mn/Fe ratio among the studied products. X-ray diffraction showed that this product was dominated by manganese-bearing oxide and silicate phases, quartz, and only minor iron-bearing components. This is consistent with its low total Fe content and with the Mössbauer spectrum, in which magnetite-type spinel components were not detected. The spectrum consisted mainly of a hematite-like component and a Fe^3+^ paramagnetic component. The hematite-like sextet with H_eff_ = 510 kOe accounted for 70% of the relative spectral area, whereas the doublet with δ = 0.33 mm/s and ΔEQ = 0.85 mm/s accounted for 30%. This doublet was assigned to a residual Fe^3+^ non-spinel component, probably related to highly dispersed or structurally disordered ferric oxide domains.

The absence of magnetite-type spinel components in the non-magnetic concentrate fraction explains the high selectivity of iron removal from this material. In contrast to the roasted ore, where spinel-type iron was detected in both separation products, the spinel-type iron formed during roasting of the concentrate was concentrated almost entirely in the magnetic fraction. The residual iron in the non-magnetic product was represented mainly by hematite-like and paramagnetic Fe^3+^ states, which have a weaker magnetic response than magnetite-type spinels.

For ferruginous manganese concentrate, magnetite-type spinel components accounted for 73% of the relative Mössbauer spectral area in the magnetic fraction. In the non-magnetic fraction, magnetite-type spinel components were not detected; therefore, this value was taken as 0%. The hematite-like component with a relative area of 24% in the magnetic fraction was not included in the calculation because it does not correspond to magnetite-type spinel phases. The Mössbauer-area-weighted fraction of iron present in magnetite-type spinel phases was calculated as follows:

$$D_{sp}=\frac{18.58\cdot 53.20\cdot 73+81.42\cdot 1.63\cdot 0}{18.58\cdot 53.20+81.42\cdot 1.63}=64.36\%$$

Thus, approximately 64.36% of iron in the roasted concentrate products was present in magnetite-type spinel phases. This value was lower than that calculated for the roasted ore; however, the separation of the concentrate was more selective because the magnetite-type spinel components were concentrated almost entirely in the magnetic fraction, whereas the non-magnetic fraction contained only residual hematite-like and paramagnetic Fe^3+^ components.

Overall, the results show that the efficiency of roasting–magnetic processing is determined not only by the total amount of magnetite-type phases formed during roasting, but also by their distribution between the magnetic and non-magnetic products. The Mn/Fe ratio reflects the technological enrichment effect, whereas Mössbauer spectroscopy explains the iron phase transformations responsible for magnetic separation. The presence of hematite-like and paramagnetic Fe^3+^ components indicates that the formation of magnetite-type spinels was incomplete under the selected roasting conditions. Therefore, the main technological limitation is not only achieving the conversion of iron-bearing phases into magnetite-type spinels, but also ensuring the preferential concentration of these spinels in the magnetic fraction.

## 4. Conclusions

Magnetization roasting followed by dry magnetic separation at 1.2 kOe promoted the selective redistribution of iron and manganese between the magnetic and non-magnetic fractions of ferruginous manganese ore and ferruginous manganese concentrate. For the roasted ferruginous manganese ore, the Mn/Fe ratio in the non-magnetic fraction increased from 3.92 to 10.87, while 76.25% of iron was transferred into the magnetic fraction and 65.86% of manganese remained in the non-magnetic product. For the roasted ferruginous manganese concentrate, the separation effect was more pronounced: the Mn/Fe ratio in the non-magnetic fraction increased from 2.98 to 23.99, 88.26% of iron was concentrated in the magnetic fraction, and 95.29% of manganese remained in the non-magnetic product.

X-ray diffraction analysis showed that the separation products are complex multiphase systems containing manganese-bearing oxide and silicate phases, quartz, hematite, and magnetite-type spinel components. However, due to the overlap of diffraction reflections of Mn–Fe oxide and spinel-type phases, X-ray diffraction alone is insufficient for resolving the iron phase states. Mössbauer spectroscopy provided additional information on the magnetic ordering and local crystallochemical environment of iron in the studied products.

Mössbauer spectroscopy revealed that the main magnetically ordered iron-bearing components associated with magnetic separation were magnetite Fe_3_O_4_ and substituted magnetite-type spinel phases of the general composition (Fe_1−x_Mx)_3_O_4_. The presence of several magnetically ordered components with different hyperfine fields indicates disturbed spinel stoichiometry and partial substitution of iron by Mn, Mg, or other cations present in the raw materials. Hematite-like α-Fe_2_O_3_ components and paramagnetic Fe^3+^/Fe^2+^ non-spinel states were also detected, indicating that the formation of magnetite-type spinels was incomplete under the selected roasting conditions.

The Mössbauer-area-weighted fraction of iron present in magnetite-type spinel phases, calculated from the relative Mössbauer spectral areas with allowance for product yield and total iron content, was 76.5% for the ferruginous manganese ore and 64.36% for the ferruginous manganese concentrate. Although this value was lower for the concentrate, its separation was more selective because the magnetite-type spinel components were concentrated almost entirely in the magnetic fraction, whereas the non-magnetic fraction contained mainly hematite-like and paramagnetic Fe^3+^ residual iron states.

The combination of X-ray diffraction and Mössbauer spectroscopy was essential for interpreting the separation products. X-ray diffraction established the crystalline oxide–silicate–spinel assemblage, whereas Mössbauer spectroscopy resolved the iron-bearing components according to magnetic ordering, valence state, and local Fe environment. This combined approach showed that the increase in the Mn/Fe ratio was governed not only by the formation of magnetite-type spinels, but also by their preferential concentration in the magnetic fractions and by the retention of residual hematite-like and paramagnetic Fe components in the non-magnetic products.

## Figures and Tables

**Table 1:** Chemical composition of the raw materials.

Material	Content, wt.%					
	Mn_total_	Fe_total_	SiO_2_	C	CaO	Mn/Fe
Mn ore	17.60	4.49	41.82	9.80	–	3.92
Mn concentrate	33.41	11.2	11.35	6.27	7.99	2.98

**Table 2:** Main characteristics of the reducing agent.

Technical Analysis *	W_t_^r^, wt.%		A^d^, wt.%			V^daf^, wt.%		S_t_^d^, wt.%			Q, kcal/kg	
	4.8		9.85			36.0		0.5			7623	
Elemental composition, wt.% **	**H^o^**			**N^o^**			**S^o^**			**O^o^_diff._**		
	5.48			1.55			0.6			14.22		
**Chemical composition of ash, wt.%**	**SiO_2_**	**Al_2_O_3_**		**Fe_2_O_3_**	**CaO**		**MgO**		**P_2_O_5_**	**K_2_O**		**Na_2_O**
	56.97	21.3		7.19	2.79		1.77		0.46	1.25		1.85
**Gas composition, vol.%**	**H_2_S**	**CO_2_**		**C_n_O_m_**	**CO**		**CH_4_**		**H_2_**	**O_2_**		**N_2_**
	1.54	22.43		0.5	14.02		28.32		29.76	0.5		2.91

Notation: * W_t_^r^—moisture; A^d^—ash content; V^daf^—volatile components; S_t_^d^—elemental sulfur. ** The superscript “o” denotes elemental composition.

**Table 3:** Yield and chemical composition of magnetic separation products obtained from roasted ferruginous manganese ore.

Product and Magnetic Field Strength	Yield, %	Content, wt.%				
		Mn_total_	Fe_total_	C	SiO_2_	Mn/Fe
Magnetic fraction at 1.2 kOe	41.40	14.51	8.27	3.08	44.25	1.75
Non-magnetic fraction at 1.2 kOe	58.60	19.78	1.82	14.54	40.10	10.87

**Table 4:** Yield and chemical composition of magnetic separation products obtained from roasted ferruginous manganese concentrate.

Product and Magnetic Field Strength	Yield, %		Content, wt.%				
		Mn_total_	Fe_total_	SiO_2_	C	CaO	Mn/Fe
Magnetic fraction at 1.2 kOe	18.58	8.50	53.2	10.08	1.56	0.87	0.16
Non-magnetic fraction at 1.2 kOe	81.42	39.10	1.63	11.64	7.35	9.61	23.99

**Table 5:** Distribution of manganese and iron between the magnetic and non-magnetic fractions.

Material	Product	Mn Distribution, %	Fe Distribution, %
Ferruginous manganese ore	Magnetic fraction	34.13	76.25
	Non-magnetic fraction	65.86	23.75
Ferruginous manganese concentrate	Magnetic fraction	4.73	88.26
	Non-magnetic fraction	95.29	11.85

**Table 6:** Mössbauer hyperfine parameters and component assignments for roasted ferruginous manganese ore.

Product	H_eff_, kOe	IS, mm/s	QS, mm/s	S, %	Component Assignment *
Magnetic fraction	487	0.26	−0.03	37	Substituted magnetite-type spinel (Fe_1−x_M_x_)_3_O_4_
	460	0.67	0.01	42	
	413	0.61	0.00	7	
	**Paramagnetic states**				
	-	1.05	0.30	5	Fe^2+^ paramagnetic component with Fe_1−x_O-like hyperfine parameters
		0.33	0.65	6	Minor Fe-bearing paramagnetic component of an unresolved non-spinel phase
		1.17	2.64	3	Minor Fe^2+^ paramagnetic component with sulfate-/silicate-like parameters
Non-magnetic fraction	487	0.18	−0.16	27	Magnetite-type spinel component Fe_3_O_4_–like
	463	0.69	0.14	19	
	**Paramagnetic states**				
	-	0.39	0.85	37	Residual Fe^3+^ non-spinel component, probably ferric oxide- or silicate-bound
	-	0.99	2.11	17	Fe^2+^ silicate-like paramagnetic component

Notation: IS, mm/s—isomer shift; QS, mm/s—quadrupole splitting; H_eff_, kOe—effective hyperfine magnetic field; S, %—relative spectral area; * M denotes a metal cation substituting for Fe in the crystal lattice.

**Table 7:** Mössbauer hyperfine parameters and component assignments for the products of roasted ferruginous manganese concentrate.

Product	H_eff_, kOe	IS, mm/s	QS, mm/s	S, %	Component Assignment *
Magnetic fraction	511	0.37	−0.18	24	Hematite-like α-Fe_2_O_3_ component
	487	0.27	−0.00	33	Substituted magnetite-type spinel (Fe_1-x_M_x_)_3_O_4_
	456	0.66	−0.00	36	
	425	0.58	−0.03	4	
	**Paramagnetic states**				
	-	0.46	0.86	3	Residual Fe^3+^ non-spinel component, probably hematite-related
Non-magnetic fraction	510	0.37	−0.19	70	Hematite-like α-Fe_2_O_3_ component
	**Paramagnetic states**				
	-	0.33	0.85	30	Residual Fe^3+^ non-spinel component, probably hematite-related

Notation: IS, mm/s—isomer shift; QS, mm/s—quadrupole splitting; H_eff_., kOe—effective hyperfine magnetic field; S, %—relative spectral area; * M denotes a metal cation substituting for Fe in the crystal lattice.

## Data Availability

The original contributions presented in this study are included in the article. Further inquiries can be directed to the corresponding author.
